# From trauma to trust: the initial psychometric evaluation of a survey instrument measuring trauma among transgender women in the US deep south

**DOI:** 10.3389/fpubh.2025.1632285

**Published:** 2025-09-04

**Authors:** Olivia T. Van Gerwen, Katelyn S. Day, Kristal J. Aaron, Hannah M. Lindl, Gabe H. Miller, D. Scott Batey, Krishmita Siwakoti, Jay Wall, Brianna Patterson, Bulent Turan, Christina A. Muzny

**Affiliations:** ^1^Division of Infectious Diseases, Department of Medicine, University of Alabama at Birmingham, Birmingham, AL, United States; ^2^Center for the Study of Sexual and Gender Health, University of Alabama at Birmingham, Birmingham, AL, United States; ^3^Heersink School of Medicine, University of Alabama at Birmingham, Birmingham, AL, United States; ^4^Department of Sociology, University of Alabama at Birmingham, Birmingham, AL, United States; ^5^School of Social Work, Tulane University, New Orleans, LA, United States; ^6^Magic City Research Institute, Birmingham AIDS Outreach, Birmingham, AL, United States; ^7^Division of Endocrinology, Department of Medicine, University of Alabama at Birmingham, Birmingham, AL, United States; ^8^Department of Psychology, Koc University, Istanbul, Türkiye

**Keywords:** trauma informed care, transgender health, sexual health, psychometric analyses, survey development

## Abstract

**Background:**

Transgender women (TGW) experience unique life traumas that may perpetuate negative sexual health outcomes, such as high rates of HIV and sexually transmitted infections. This is especially true in the US Deep South, where structural and cultural factors further marginalize gender minorities as well as people of color. Providing trauma informed care to TGW in sexual and reproductive health (SRH) settings is necessary, but strategies to measure traumatic experiences among this population are needed. We aimed to develop and psychometrically assess a multi-item survey instrument evaluating trauma-specific histories for use with TGW in SRH settings and assess differences in reported trauma histories between White and non-White TGW in the US Deep South.

**Methods:**

Survey items were developed using three existing general trauma instruments (Life Events Checklist for DSM-5, Trauma History Questionnaire, Stressful Life Events Screening Questionnaire) and results from qualitative interviews with TGW. Survey items fell into five trauma subdomains: healthcare-related experiences, sexual/relationship experiences, crime-related/general trauma experiences, gender dysphoria experiences, and discrimination experiences. A computer-assisted self-interviewing instrument was administered to TGW. Descriptive statistics were calculated. Cronbach’s alpha coefficients (*α*) were calculated for each subdomain to determine internal consistency. Results were stratified by race (White versus non-White), and means of trauma subdomain results were compared.

**Results:**

Between April 2024–September 2024, 105 TGW enrolled and completed the instrument. Median participant age was 30 years (range 19–73), and most identified as White (*n* = 55) or Black/African American (*n* = 40). Mental health conditions such as depression (*n* = 64) and anxiety (*n* = 59) were common. Psychometric analyses revealed acceptable internal constancy for the subdomains of healthcare-related experiences (*α* = 0.787), crime-related/general trauma experiences (*α* = 0.870), and discrimination experiences (α = 0.870). Subdomains measuring sexual/relationship experiences and gender dysphoria had lower reliability (α = 0.597 and 0.499, respectively). Trauma in all subdomains was common among all participants, with traumatic sexual and relationship experiences (*p* = 0.004) and crime-related and general trauma experiences (*p* < 0.001) reported more frequently among non-White participants and gender dysphoria experiences (*p* < 0.001) reported more frequently among White participants.

**Conclusion:**

TGW experience trauma in multiple domains, and the intersection of race and gender minority status appears to influence these findings. This instrument has the potential to facilitate trauma assessment in SRH clinical settings and embolden providers to provide care through a trauma informed lens.

## Introduction

Approximately 1.4 million people in the United States (US) identify as transgender and almost one-third of those identify as transgender women (TGW) (1). This population experiences a dramatic burden of mental health conditions including depression, anxiety, and post-traumatic stress disorder (PTSD), which is associated with a variety of traumatic experiences (2, 3). In previous qualitative work, such trauma has been noted to impact TGW in a pervasive, multi-level manner with prominent themes including barriers to healthcare, anti-transgender legislation, misgendering/deadnaming experiences, limited social network support, violence, stigma, and dysphoria (4). These traumas are particularly significant in the healthcare setting (5, 6), which provides some explanation for the limited healthcare engagement by TGW and for some of the health disparities they experience (7). Specific to sexual and reproductive health (SRH), TGW are disproportionately affected by HIV and sexually transmitted infections (STIs) when compared to cisgender people and, therefore, would benefit from SRH services tailored to their needs (8–10).

Trauma-informed care (TIC) is an approach that acknowledges the profound effects trauma can have on a person’s overall well-being, aiming to prevent re-traumatization as patients utilize healthcare services (11). When utilized by healthcare professionals in SRH settings, TIC has the potential to improve engagement with healthcare services as well as sexual health outcomes of TGW. In addition to appreciating the specific traumas of one’s patient population, having tools to assess those traumas is also essential to providing TIC. Currently, no validated trauma measurement instrument exists specific to TGW. Such an instrument would provide a crucial tool for clinicians in SRH settings as it could guide them in mitigating some of the negative experiences TGW may face in these settings (e.g., invasive examinations, unearthing of previous trauma, exacerbation of gender dysphoria), with the goal of better patient experiences and higher engagement with SRH services.

Using the results of our previous qualitative work (4) and existing trauma instruments validated in the general population (12–14), we aimed to develop a multi-item survey instrument assessing trauma-specific histories for use among TGW in SRH settings. Here, we report the development and initial psychometric assessment of this survey instrument in addition to our results from administering the instrument to a cohort of TGW in the US Deep South. Such work is particularly important in this region given the confluence of transphobic cultural dynamics that manifest in healthcare settings (15), high rates of HIV and STIs (16, 17), and poor mental health outcomes when compared to other parts of the US (18, 19). We also looked for differences between White and non-White participants in terms of reported trauma experiences. Given the effect of intersecting racial and gender minority identities on trauma, we hypothesized that non-White TGW would endorse more trauma experiences than White TGW.

## Methods

### Survey instrument development

To inform the development of the survey instrument, we enrolled 13 TGW to participate in qualitative in-depth interviews where they described lived experiences of trauma, both individually and among members of their community (results reported elsewhere) (4). These participants were recruited through flyers posted in community spaces (e.g., bars, restaurants, nail salons, clubs) in the Birmingham, Alabama metropolitan area as well as through partnerships with local LGBTQ+ servicing organizations and clinics. Recruitment methods included word-of-mouth referral, snowball sampling, and social media messaging. Participants met with experienced qualitative researchers and were asked to share their lived experiences of trauma as TGW in the US Deep South as well as the role that SRH plays in that experienced trauma. The research team then developed measures relevant to the common themes of trauma elucidated from these interviews.

### Survey instrument items

**Figure 1:**
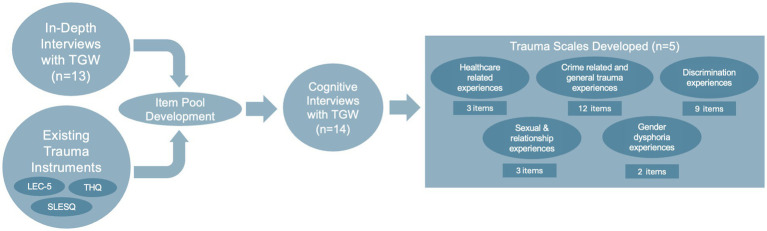
Process of survey development. LEC-5, Life Events Checklist for DSM-5; SLESQ, Stressful Life Events Screening Questionnaire; THQ, Trauma History Questionnaire; TGW, transgender women.

All trauma-related items in the survey were adapted from themes elucidated from in-depth interviews (4) as well as three existing, publicly available validated trauma instruments developed for the general population: the Life Events Checklist for DSM-5 (LEC-5) (14), the Trauma History Questionnaire (THQ) (12), and the Stressful Life Events Screening Questionnaire (SLESQ) (13). Some items required a response on a frequency scale of an experienced trauma (i.e., “never,” “once,” “2–3 times,” “4 or more times”) while others included “yes” or “no” responses, with some items requiring a follow up question (usually free text) if the answer was “yes.” The survey instrument contained some additional items that allowed participants to further characterize an experience, typically by providing free text responses. These components were not considered in the psychometric analyses of the survey instrument. Similar items were grouped together in five different subdomains (healthcare related experiences, sexual and relationship experiences, crime related and general trauma experiences, gender dysphoria experiences, and discrimination experiences) to capture information about similar types of traumatic experiences. These domains are summarized in Table 1, and the survey instrument development process is shown visually in Figure 1. The full survey that was administered to participants can be found in Appendix 1.

**Table 1:** Trauma scales, questions, and response options.

Scales and questions	Response options
*Healthcare-Related Experiences (3 items)* How many times have you felt mistreated by a medical professional while receiving care, because of your gender identity? How many times have you feared for your physical safety when you have been in a healthcare setting? How many times have you feared for your mental/emotional wellbeing when you have been in a healthcare setting?	Never Once 2–3 times 4 or more times
*Sexual and Relationship Experiences (3 items)* Has anyone made you have intercourse or oral or anal sex against your will? Have you participated in transactional sex (also known as sex work, prostitution, etc.)? Have you been in a relationship (e.g., emotional, sexual, romantic) with someone where you felt there were unequal power dynamics (i.e., one person has more power in the relationship than the other)?	Yes No
*Crime-Related and General Trauma Experiences (12 items)* Has anyone attempted to rob or actually robbed you? Have you been arrested or incarcerated? Do you feel that you have experienced poor treatment by law enforcement services or while incarcerated? Have you seen someone seriously injured or killed? Have you had a spouse, romantic partner, child, or other loved one die? Have you have had a serious or life-threatening illness? Has anyone attacked you with a gun, knife, or some other weapon? Has anyone attacked you without a weapon? Have you ever experienced homelessness? Have you ever experienced food insecurity? Have you ever experienced unemployment or dire financial struggles?	Yes No
*Gender Dysphoria Experiences (2 items)* Do you experience dysphoria around your sex assigned at birth? Does someone misgendering you give you dysphoria?	Yes No
*Discrimination Experiences (9 items)* How often have you experienced discrimination at school? How often have you experienced discrimination getting hired or getting a job? How often have you experienced discrimination at work? How often have you experienced discrimination getting housing? How often have you experienced discrimination getting medical care? How often have you experienced discrimination getting services in a store or restaurant? How often have you experienced discrimination getting credit, bank loans, or mortgage? How often have you experienced discrimination on the street or in a public setting? How often have you experienced discrimination from the police or in the courts?	Never Once 2–3 times 4 or more times

After initial development, the survey instrument was then tested with 14 TGW using cognitive interviews (20, 21). Cognitive interviewing is a qualitative research method which involves asking participants to review survey materials and provide insights and feedback into how they interpret questions and is a commonly used validation tool in survey development (22, 23). The research team reviewed feedback obtained during the cognitive interviews and further refined the items of the instrument before creating a web-based version of the instrument in Qualtrics that could be administered to study participants.

### Participants, settings, and procedures

TGW were recruited from both community and clinical sites in Birmingham, AL to complete the survey instrument. A variety of community-engaged strategies were implemented to engage the local TGW community including administering the survey on tablets during clinic visits at the University of Alabama at Birmingham (UAB) Gender Health Clinic, an in-service presentation and enrollment event at a local advocacy organization run by and serving Black TGW, and a text messaging campaign inviting patients of a local LGBTQ+ community health center to participate. While recruitment efforts were based in Birmingham, the clinics and orgnizations involved in this study have wide catchment areas that serve the state of Alabama broadly, including rural, urban, and suburban communities. Recruitment took place from April 2024 to September 2024. Individuals were eligible for the study if they were 18 years or older, English-speaking, and self-identifying as a TGW or any other transfeminine gender identity.

This study protocol was approved by the UAB Institutional Review Board (Protocol #300009382). This approval was granted with the sensitive nature of data and vulnerability of participants in mind. In order to protect confidentiality and anonymity, potential participants were assigned a unique code preventing linkage of survey data to any individual, and they could either complete the survey in-person or on a tablet or be sent an anonymous link to the survey to complete on their own mobile device. Further, signed consent was waived to avoid participants being required to affix their name to any study documents; instead, participants reviewed an information sheet about the study and consented to participate by clicking an electronic check box. Participants were informed that they could decline to answer any survey item without explanation, and they could decide to end their study participation at any time without consequence. In the event that survey questions caused undue psychological or emotional stress, immediate referral to licensed mental health providers was available at study sites to intervene with participants. The survey instrument and additional socio-demographic questions were subsequently administered to participants who agreed to participate in the study. Participants were compensated with $20 for completing the survey.

### Data management and analysis

Data were collected and stored in a secure, HIPAA-compliant Qualtrics database (Qualtrics, Provo, UT). Descriptive statistics for the sample were calculated. This included the overall sociodemographic characteristics of the sample as well as the results of the five trauma subdomains. Sociodemographic questions included details about race, ethnicity, age, insurance status, substance use history, sexual orientation, genders of sexual partners, and experience with gender-affirming care (e.g., gender-affirming hormone therapy, gender-affirming surgical procedures). For the healthcare related experiences and discrimination experiences subdomains, response options range from “never” (scored as 0) to “4 or more times” (scored as 3). Items are summed for a score range of 0 to 9 for the healthcare related experiences subdomain and 0 to 27 for the discrimination experiences subdomain. For the subdomains of sexual and relationship experiences, crime-related and general trauma experiences, and gender dysphoria experiences, response options were dichotomous with ‘No’ (scored as 0) and ‘Yes’ (scored as 1). For the sexual and relationship experiences subdomain, the items summed for a score range of 0 to 3. For the crime-related and general trauma experiences subdomain, the items summed for a score range of 0 to 12 and for the gender dysphoria experiences subdomain, the items summed for a score range of 0 to 2. Higher scores indicated more negative experiences for each subdomain. Cronbach’s alpha coefficients were calculated for each subdomain to determine internal consistency. The means and standard deviation were determined by dividing the sum of each subdomain by the number of items. Race was dichotomized into White versus non-White (Black/African-American, Native American, and Multiple races) for stratified analyses. To compare the means of the subdomains by race, the Mann–Whitney U test was employed due to the non-normal distribution of the data. To account for multiple subdomains within the survey instrument (*n* = 5), we input raw *p*-values and used proc. multtest to adjust *p*-values for multiple comparisons using the stepdown Bonferroni correction. Additionally, items under the healthcare related experiences and discrimination experiences subdomains were dichotomized to reflect whether participants had experienced a given situation at least once (scored as 1) versus none (scored as 0). Items under sexual and relationship experiences, crime-related and general trauma experiences, and gender dysphoria experiences were already dichotomized. The number and frequency for each item under each subdomain was calculated for descriptive purposes only. All analyses were performed using SAS 9.4 (Cary, NC, United States).

## Results

### Study participant characteristics

Between April and September 2024, 105 TGW enrolled and completed the instrument. The median age was 30 years (range 19–73 years). Most participants identified as White (*n* = 55, 52.9%) or Black/African American (*n* = 40, 38.1%). Most participants had health insurance (*n* = 94, 89.5%). The most frequently reported illicit substances used (lifetime) included marijuana (*n* = 63, 60.0%), crack or powder cocaine (*n* = 28, 26.6%), and hallucinogenic drugs (*n* = 20, 19.0%); 29 participants (27.6%) reported no history of illicit substance use. Mental health conditions were commonly reported, with 85 participants (81.0%) designating that they had at least one diagnosis. Depression (*n* = 64, 61.0%) and anxiety (*n* = 59, 56.2%) were reported as the most common mental health diagnoses. Participants reported a variety of sexual orientations and genders of sexual partners. Among our sample, most participants reported being on gender affirming hormone therapy at the time of survey completion (*n* = 79, 75.2%), but most reported no history of gender affirming surgeries or procedures (*n* = 82, 85.4%). Table 2 summarizes the demographic characteristics of this sample.

**Table 2:** Descriptive statistics for a sample of transgender women in Birmingham, AL (*N* = 105).

Characteristic	Median (range) or N (%)
Age (years), range	30 (19–73)
Race	
White	55 (52.9%)
Black/African American	40 (38.1%)
Native American	1 (1.0%)
Multiple races	6 (5.7%)
Ethnicity	
Hispanic/Latino	5 (4.8%)
Not Hispanic/Latino	82 (78.1%)
Highest Level of Education	
Less than high school	11 (10.5%)
High school graduate/GED	36 (34.3%)
Some college/Assoc degree	33 (31.4%)
4-year degree/Bach degree	17 (16.2%)
Any post-graduate studies	5 (4.8%)
Occupational status	
Employed part or full-time	51 (48.6%)
Student, employed part or full-time	4 (3.8%)
Student only	4 (3.8%)
Student on disability	1 (1.0%)
Disabled	11 (10.5%)
Unemployed	31 (29.5%)
Relationship status	
Married	12 (11.4%)
In a relationship	16 (15.2%)
Separated or divorced	16 (15.2%)
Single/Never married	55 (52.4%)
Health insurance status	
Insured	94 (89.5%)
Private	53 (56.4%)
Public (Medicare, Medicaid, VA)	38 (40.4%)
Tricare	3 (3.2%)
Uninsured	11 (10.5%)
Alcohol use	
Never	32 (30.5%)
Monthly or less	33 (31.4%)
2–4 times per month	35 (33.3%)
2–3 times per week	4 (3.8%)
4 or more times per week	1 (1.0%)
Lifetime illicit/recreational drug use*	
None	29 (27.6%)
Marijuana/hash	63 (60.0%)
Amphetamine/Methamphetamine/Crystal meth	12 (11.4%)
Crack	12 (11.4%)
Powder cocaine	16 (15.2%)
Heroin	6 (5.7%)
Hallucinogenic drugs	20 (19.0%)
Prescription drugs in a way not prescribed	15 (14.3%)
Current mental health diagnoses*	
None	17 (16.2%)
Anxiety	59 (56.2%)
Depression	64 (61.0%)
Bipolar Disorder	28 (26.7%)
ADD/ADHD	35 (33.3%)
Schizophrenia/Schizoaffective	8 (7.6%)
PTSD	20 (19.0%)
Other mental health issue	7 (6.7%)
Sexual Orientation*	
Heterosexual	17 (16.2%)
Homosexual	32 (30.5%)
Bisexual	26 (24.8%)
Asexual	1 (1.0%)
Queer	22 (21.0%)
Other/multiple/not listed	17 (16.2%)
Genders of sexual partners*	
Cis men	50 (47.6%)
Cis women	30 (28.6%)
Trans men	12 (11.4%)
Trans women	50 (47.6%)
Not sexually active	2 (1.9%)
Non-binary, Genderfluid, Gender diverse	22 (21.0%)
Other/multiple	2 (1.9%)
Currently on HRT	
Yes	79 (75.2%)
No	26 (24.8%)
History of gender affirming procedures*	
None	82 (85.4%)
Breast augmentation	6 (5.7%)
Facial feminization surgery	5 (4.8%)
Vocal cord procedures	1 (1.0%)
Orchiectomy	1 (1.0%)
Vaginoplasty	2 (1.9%)
Other procedures	5 (4.8%)

*Participants could select more than one for these variables, so N(%) will not add up to the total N (100%). Data for age was missing for 16.2% of participants. Data for ethnicity was missing for 17.1% of participants. Data for race, education, occupational status, current and lifetime illicit drug use, current mental health diagnosis, and sexual orientation was missing for 2.9% of participants. Data for relationship status was missing for 5.7% of participants. Data for the gender of sexual partners was missing for 6.7% of participants. Data on the history of gender affirming care was missing for 8.6% of participants.

### Psychometric analyses

The subdomains of healthcare-related experiences, crime-related/general trauma experiences, and discrimination experiences all showed acceptable internal consistency (Cronbach’s *α* = 0.787, 0.870, and 0.870, respectively) after dichotomization. However, subdomains measuring sexual/relationship experiences and gender dysphoria had lower reliability (Cronbach’s α = 0.597 and 0.499, respectively). These results are displayed in Table 3.

**Table 3:** Trauma instrument reliability.

Scale	Number of items	Cronbach’s α
Healthcare-related experiences	3	0.787
Sexual and relationship experiences	3	0.597
Crime-related and general trauma experiences	12	0.870
Gender dysphoria experiences	2	0.499
Discrimination experiences	9	0.870

### Outcomes

The means of the subdomains of the instrument, stratified by race, for all participants are shown in Table 4. For descriptive purposes, the individual items of each subdomain, stratified by race, are presented in Supplementary Table 1.

**Table 4:** Results of the trauma survey administered to TGW, stratified by self-reported racial identity.

Variable	Total	White	Non-white	*p*-value
Healthcare-related experiences (mean ± SD)	0.75 ± 0.82	0.85 ± 0.81	0.61 ± 0.81	0.163
Sexual and relationship experiences (mean ± SD)	0.44 ± 0.36	0.35 ± 0.36	0.55 ± 0.34	0.011*
Crime-related and general trauma experiences (mean ± SD)	0.47 ± 0.31	0.37 ± 0.25	0.59 ± 0.34	0.002*
Gender dysphoria experiences score (mean ± SD)	0.80 ± 0.32	0.93 ± 0.2	0.64 ± 0.38	0.001*
Discrimination experiences score (mean ± SD)	0.80 ± 0.74	0.65 ± 0.62	0.96 ± 0.84	0.163

*Healthcare-Related Experiences.* There were no statistically significant differences between White and non-White participants for the mean scores for this subdomain (*p* = 0.163). Among participants, over half reported having experienced mistreatment while receiving healthcare (*n* = 53, 51.4%) and having feared for their mental or emotional wellbeing in a healthcare setting (*n* = 52, 49.5%) because of their gender identity at least once (Supplementary Table 1).

*Sexual and Relationship Experiences.* There was a statistically significant difference in subdomain mean scores between White (mean score = 0.35 ± 0.36) versus non-White (mean score = 0.55 ± 0.34) participants (*p* = 0.011). Among participants, a history of forced sexual intercourse (*n* = 35, 33.3%), lifetime participation in transactional sex (*n* = 44, 41.9%), and having been in a relationship characterized by unequal power dynamics (*n* = 65, 58.0%) were common experiences. In our sample, non-White participants reported a greater frequency of participation in transactional sex (non-White *n* = 28 [56.0%] versus White *n* = 16 [29.1%]) (Supplementary Table 1).

*Crime-Related and General Trauma Experiences.* There was a statistically significant difference in subdomain mean scores between White (mean score = 0.37 ± 0.25) versus non-White (mean score = 0.59 ± 0.34) participants (*p* = 0.002). All items within this subdomain were frequently reported among participants, but the three most prevalent were experiencing unemployment or financial struggles (*n* = 70, 66.7%), having a serious or life-threatening illness (*n* = 63, 60.0%), or having been abandoned, disowned, or estranged by members of their biological family (*n* = 60, 57.1%). Non-White participants more frequently reported the following compared to White participants: experiencing robbery or attempted robbery (non-White *n* = 25 [50.0%] versus White *n* = 7 [12.7%]), having been arrested or incarcerated (non-White *n* = 24 [48.0%] versus White *n* = 12 [21.8%]), having been attacked with a gun/knife/other weapon (non-White *n* = 27 [54.0%] versus White *n* = 7 [12.7%]), and experiencing homelessness (non-White *n* = 36 [72.0%] versus White *n* = 10 [18.2%]; Supplementary Table 1).

*Gender Dysphoria Experiences*. There was a statistically significant difference in subdomain mean scores between White (mean score = 0.93 ± 0.2) versus non-White (mean score = 0.64 ± 0.38) participants (*p* = 0.001). Experiences with gender dysphoria associated with sex assigned at birth (*n* = 87, 83.7%) and being misgendered (*n* = 79, 76.7%) were common among study participants. White participants more commonly reported experiencing gender dysphoria associated with their sex assigned at birth compared to non-White participants (White *n* = 54 [98.2%] versus non-White *n* = 33 [67.4%]; Supplementary Table 1).

*Discrimination Experiences.* There were no statistically significant differences between White and non-White participants for the mean scores for this subdomain (*p* = 0.163). The three most commonly endorsed items were experiencing discrimination on the street or in a public setting (*n* = 70, 66.7%), at work (*n* = 58, 55.8%), or when getting hired for a job (*n* = 49, 46.7%) on the basis of gender identity. Non-White participants more frequently reported experiencing discrimination related to getting housing at least once compared to White participants (White *n* = 5 [9.1%] versus non-White participants (*n* = 20 [40%]; Supplementary Table 1).

## Discussion

The results of this survey administered to a diverse sample of TGW in the US Deep South demonstrate the multiple domains in which trauma inundates their daily lives. Discrimination, sexual trauma, and mistreatment in a myriad of settings were reported by participants frequently. These findings support existing data reporting a high burden of psychological trauma among TGW and provide a striking glimpse into the areas of their lives that are most impacted (6). Healthcare providers and all members of social support and care teams (e.g., social workers, community health workers) must consider these lived experiences when delivering care and engaging with this patient population. This survey instrument provides an essential tool that can be used to assess such experiences in SRH settings. The variety of racial backgrounds, sexual orientations, mental health histories, and other demographic factors of the sample supports the survey’s usefulness and validity among a diverse array of TGW.

Three of the five subdomains (healthcare-related experiences, crime-related/general trauma experiences, discrimination experiences) demonstrated acceptable internal consistency, indicating that the items within the subdomain are highly related to each other and, taken together, consistently measure their intended construct (24). The other two subdomains (gender dysphoria experiences, sexual and relationship experiences) demonstrated suboptimal internal consistency, with the most likely explanation for this being the small number of items included in the subdomains. Despite this limitation, the responses to the individual items for these subdomains could be useful clinically for healthcare providers, particularly with regards to understanding how individual TGW patients have experienced sexual assault, rape, transactional sex, dysphoria, and misgendering. The data generated by the administration of this survey also emphasizes the profound impact gender dysphoria and sexual trauma have on TGW.

As hypothesized, our sample had variable experience with certain types of traumas when stratified by race. Non-White participants reported experience with transactional sex significantly more than White participants. It is well described that TGW in general have higher rates of participation in transactional sex than other populations, largely due to systemic factors that force them into such work including poverty and structural transphobia (25). The intersection of race and gender are evident in this case, where structural factors negatively impacting TGW of color are amplified, often leading to the need for participation in transactional sex for basic survival. This disparity could portent worse sexual health outcomes (e.g., higher likelihood of HIV/STI acquisition, risk of sexual assault) for individuals multiple minority identities, such as TGW of color. The implications of more frequent sex work among TGW of color are important to consider, especially in SRH settings. Tailored sexual health screening and prevention offerings are key in serving this subpopulation.

Along similar lines, crime-related experiences were more commonly reported among non-White participants, again emphasizing the role intersecting racial and gender identities play on the experience of trauma among TGW. While these experiences were higher among TGW of color, they were relatively common among all participants. This highlights the need for safe SRH care environments and health systems at large that consider and accommodate for the experienced trauma of this population that can facilitate their engagement in care, particularly among ethnic and racial minorities.

Gender dysphoria experiences were more common among White participants than those who were not White. Prior to answering questions in this subdomain, participants were provided with a definition of gender dysphoria (“a sense of unease that a person may have because of a mismatch between their sex assigned at birth and their gender identity”) so cultural differences around use of this term was accounted for. In addition, the cognitive interviews that were conducted in preparation of survey development included a review of the provided definition to a racially diverse sample of TGW. Despite these efforts, it is still possible that participants did not review the provided definition, and, even if they did, their conceptual understanding of the definition may still have been limited. The reasons for racial differences among our sample related to experiencing gender dysphoria and being misgendered are unclear, but it is possible that there are cultural differences between how White and non-White TGW perceive their gender identity that are contributing.

This study is not without limitations. This survey was administered to a convenience sample of TGW in the US Deep South, with a large number of those being recruited through clinics and advocacy organizations. The lived experiences of this sample may not reflect those of people from other parts of the US and globally as well as the most marginalized and underrepresented in the US Deep South, limiting generalizability. Further validation of this instrument should include sites from other regions. As noted above, the low internal consistency of two of the subdomains in this survey limit the ability to use the subdomain in and of itself as a measure of those particular trauma constructs. Another limitation is that only internal consistency was measured in this study and further exploration of factor structure was not undertaken. Future validation efforts will focus on conducting assessments of convergent, discriminant, and predictive validity to enhance the clinical utility of this instrument. Regardless, the individual questions posed by these two sections of the survey still offer clinical utility to providers in SRH who seek to better understand their patients’ trauma history while providing care.

Despite these limitations, this novel survey instrument shows promise for use in SRH clinical settings in the Deep South. Future directions should include investigating ways to more rigorously capture trauma-related data in the gender dysphoria and sexual/relationship subdomains. This could include developing further questions through community engaged inquiry and integrating them into the instrument. Further exploration of impacts of additional sociodemographic factors, such as rural versus urban status and regilious affiliation, could also provide further context to the interpretation of traumatic experiences among TGW. Additional work also needs to be done to determine how clinicians can use scores for each subdomain to clinically assess TGW patients in a meaningful manner.

## Conclusion

The results of this study elevate the experiences of TGW in the US Deep South and provide insights into the trauma landscape that influences their daily lives. In addition to illuminating the variety and frequency of traumatic experiences among our sample and differences between White and non-White TGW, the survey demonstrated favorable psychometric characteristics of the subdomains measuring healthcare-related experiences, crime-related/general trauma experiences, and discrimination experiences. The other two subdomains (gender dysphoria experiences, sexual and relationship experiences) also provided important and clinically useful information for clinicians aiming to provide trauma informed SRH. These findings underscore the survey’s potential utility in addressing the unique needs of TGW populations in a trauma informed manner to improve their care and engagement in SRH settings and beyond.

## Data Availability

The raw data supporting the conclusions of this article will be made available by the authors, without undue reservation.
