# Supplementary material for: From trauma to trust: the initial psychometric evaluation of a survey instrument measuring trauma among transgender women in the US deep south
Source: Front Public Health. 2025 Sep 4;13:1632285. doi: 10.3389/fpubh.2025.1632285 (PMC12443781; doi:10.3389/fpubh.2025.1632285)
Supplement: Supplementary file 2 [file Table_1.docx]

**Supplemental Table 1.**

| Variable | Total | White | Non-White |
| --- | --- | --- | --- |
| Healthcare-Related Experiences |  |  |  |
| Felt mistreated by a medical professional while receiving care because of gender identity at least once (n=103) | 53 (51.4%) | 30 (56.6%) | 23 (46.0%) |
| Feared for physical safety when in a healthcare setting at least once (n=103) | 30 (29.1%) | 15 (28.3%) | 15 (30.0%) |
| Feared for mental/emotional wellbeing in a healthcare setting at least once (n=105) | 52 (49.5%) | 34 (61.8%) | 18 (36.0%) |
| Sexual and Relationship Experiences |  |  |  |
| Had intercourse or oral or anal sex against your will (n=105) | 35 (33.3%) | 14 (24.5%) | 21 (42%) |
| Has ever participated in transactional sex (n=105) | 44 (41.9%) | 16 (29.1%) | 28 (56.0%) |
| Has been in a relationship with someone where there were unequal power dynamics (n=105) | 65 (58.0%) | 28 (50.9%) | 33 (66.0%) |
| Crime-Related and General Trauma Experiences |  |  |  |
| Has experienced robbery or attempted robbery (n=105) | 32 (30.4%) | 7 (12.7%) | 25 (50.0%) |
| Has been arrested or incarcerated (n=105) | 36 (34.3%) | 12 (21.8%) | 24 (48.0%) |
| Has experienced poor treatment by law enforcement or while being incarcerated (n=105) | 35 (33.3%) | 14 (25.5%) | 21 (42%) |
| Has seen someone seriously injured or killed (n=104) | 50 (48.1%) | 24 (43.6%) | 26 (53.1%) |
| Has had a spouse, romantic partner, child or other loved one die (n=103) | 58 (56.3%) | 27 (50.0%) | 31 (63.3%) |
| Has had a serious or life-threatening illness (n=105) | 63 (60.0%) | 32 (58.2%) | 31 (62.0%) |
| Has been attached with a gun, knife, or some other weapon (n=105) | 34 (32.4%) | 7 (12.7%) | 27 (54.0%) |
| Has been attacked without a weapon (n=105) | 52 (49.5%) | 24 (43.6%) | 28 (56.0%) |
| Has experienced homelessness (n=105) | 46 (43.8%) | 10 (18.2%) | 36 (72.0%) |
| Has experienced food insecurity (n=105) | 56 (53.3%) | 25 (45.5%) | 31 (62.0%) |
| Has experienced unemployment or dire financial struggles (n=105) | 70 (66.7%) | 31 (56.4%) | 39 (78.0%) |
| Has been abandoned, disowned, or estranged by members of biological family (n=105) | 60 (57.1%) | 29 (52.7%) | 31 (62%) |
| Gender Dysphoria Experiences |  |  |  |
| Experiences dysphoria around sex assigned at birth (n=104) | 87 (83.7%) | 54 (98.2%) | 33 (67.4%) |
| Experiences dysphoria with misgendering (n=103) | 79 (76.7%) | 48 (87.3%) | 31 (64.6%) |
| Discrimination Experiences |  |  |  |
| Has experienced discrimination at school at least once (n=105) | 48 (45.7%) | 20 (36.4%) | 28 (56.0%) |
| Has experienced discrimination in getting hired for a job at least once (n=105) | 49 (46.7%) | 23 (41.8%) | 26 (52.0%) |
| Has experienced discrimination at work at least once (n=104) | 58 (55.8%) | 29 (52.7%) | 29 (59.2%) |
| Has experienced discrimination getting housing at least once (n=105) | 25 (23.8%) | 5 (9.1%) | 20 (40.0%) |
| Has experienced discrimination getting medical care at least once (n=104) | 31 (29.8%) | 16 (29.6%) | 15 (30.0%) |
| Has experienced discrimination getting services in a store or restaurant at least once (n=105) | 40 (38.1%) | 15 (27.3%) | 25 (50.0%) |
| Has experienced discrimination getting credit, bank loans, or mortgage at least once (n=104) | 14 (13.5%) | 3 (5.5%) | 11 (22.5%) |
| Has experienced discrimination on the street or in a public setting at least once (n=105) | 70 (66.7%) | 35 (63.6%) | 35 (70.0%) |
| Has experienced discrimination from the police or in the courts at least once (n=105) | 34 (32.4%) | 12 (21.8%) | 22 (44.0%) |
