# Supplementary material for: From trauma to trust: the initial psychometric evaluation of a survey instrument measuring trauma among transgender women in the US deep south
Source: Front Public Health. 2025 Sep 4;13:1632285. doi: 10.3389/fpubh.2025.1632285 (PMC12443781; doi:10.3389/fpubh.2025.1632285)
Supplement: Supplementary file 2 [file Supplementary_file_1.docx]

Trauma informed care among transgender women in the Southeastern U.S.

Start of Block: Input required by study personnel

Q1 Study ID:

________________________________________________________________

|  |  |
| --- | --- |

Q2 Date form completed:

________________________________________________________________

End of Block: Input required by study personnel

Start of Block: Consent

Q3 **Title of Research:** Measuring traumatic experiences and their impact on sexual health behaviors and care utilization among transgender women in the Southeastern U.S (Survey)

**UAB IRB Protocol #:** IRB-300009382

**Principal Investigator:** Olivia Van Gerwen, MD, MPH

                                       Zeigler Research Building, Rm 218A

                                       703 19th Street South Birmingham, AL 35294

**Study Related Phone Numbers:** Olivia Van Gerwen, MD, MPH 205-644-1273

                                                       205-934-BEEP (2337); Enter Pager #: 7045 (24-hour pager)

**Sponsor:** UAB Department of Medicine

**Purpose of the Research Study**

The University of Alabama at Birmingham (UAB) invites you to participate in a research study. We are asking you to take part in this research study because you are a transgender woman who meets all the study’s eligibility criteria. The purpose of this research study is test the performance of a survey our team has developed. This survey aims to collect information about the traumatic experiences patients have encountered in your community that would be helpful for your healthcare provider to know in a clinical setting. We are also asking questions about your sexual health, sexual behaviors, and engagement with the health care system to try and understand how trauma impacts these factors. We will enroll 100 transgender women in Birmingham, Alabama to participate in this survey.

**Study Participation & Procedures**

If you decide to participate in this study, you will complete an electronic survey on a tablet device. This survey is being offered to participants in clinical settings such as the UAB Gender Health Clinic but may also be administered remotely or in the community. The survey will be offered by a member of our research team and then you will be given a tablet, on which you will review this information sheet and fill in a check box if you wish to proceed. After that, you will scroll through the survey on the tablet on your own (a research team member will be present in the room in case you need technical assistance, but they will not be asking you the questions) and answer the survey questions. This should take no more than 30 mintutes to complete. Your answers will be stored in a secure, online database, for which only our research team will have access to. We will not be collecting any identifiable information such as your name, address, or other contact information as part of this survey.

**Risks and Discomforts**

The study will involve answering questions related to trauma, mental health, sexual health, sexually transmitted infections (STIs), and HIV prevention among transgender women, which may cause some discomfort. You are welcome to decline to answer any question at any time or end the survey early at any point if you do not wish to continue.

**Benefits**

Your answers to the questionnaire items will help our team to refine this survey instrument and to better understand how to provide trauma-informed care to transgender women.

**Alternatives**

Your alternative is to not participate in the study.

**Confidentiality**

Information obtained about you for this study will be kept confidential to the extent allowed by law. However, research information that identifies you may be shared with people or organizations for quality assurance or data analysis, or with those responsible for ensuring compliance with laws and regulations related to research. They include:

• the UAB Institutional Review Board (IRB). An IRB is a group that reviews the study to protect the rights and welfare of research participants.

• UAB School of Medicine

• the Office for Human Research Protections (OHRP)

The information from the research may be published for scientific purposes; however, your identity will not be given out in those publications.

**Voluntary Participation and Withdrawal**

Whether or not you take part in this study is your choice. There will be no penalty if you decide not to be in it. If you decide not to be in the study, you will not lose any benefits you are otherwise owed. You are free to withdraw from this study at any time. Your choice to leave the study will not affect your relationship with this institution. Contact the study Principal Investigator if you want to withdraw from the study. You may be removed from the study without your consent if the sponsor ends the study, if the Principal Investigator decides it is not in the best interest of your health, or if you are not following the study rules. If you are a UAB student or employee, taking part in this research is not a part of your UAB class work or duties. You can refuse to enroll or withdraw after enrolling at any time before the study is over, with no effect on your class standing, grades, or job at UAB. You will not be offered or receive any special consideration if you take part in this research.

**Cost of Participation**

There is no cost to you for taking part in this study. UAB will pay for all study materials used during your interview session.

**Payment for Participation** 

You will be paid $20 for completing this survey. Payments will be made by ClinCard, a reloadable payment card, which will be given to you during your study visit today if you do not already have a ClinCard.

**Payment for Research-Related Injuries**

UAB will not provide any payment if you are harmed as a result of taking part in this study. If such harm occurs, treatment will be provided. However, this treatment will not be provided free of charge.

**Contact for Questions or Concerns**

If you have any questions, concerns, or complaints about the research or a research-related injury including available treatments, please contact the study Principal Investigator. You may contact Dr. Olivia Van Gerwen at 205-644-1273 or after hours by paging her at the following pager number: 205-934-BEEP (2337); Enter Pager 7045 (24-hour pager). If you have questions about your rights as a research participant, or concerns or complaints about the research, you may contact the UAB Office of the IRB (OIRB) at (205) 934-3789 or toll free at 1-855-860-3789. Regular hours for the OIRB are 8:00 a.m. to 5:00 p.m. CT, Monday through Friday.

**Legal Rights**

You are not waiving any of your legal rights by signing this consent form.

**CONFIDENTIALITY AND AUTHORIZATION TO USE AND DISCLOSE INFORMATION FOR RESEARCH PURPOSES** 

Federal regulations give you certain rights related to your health information. These include the right to know who will be able to get the information and why they may be able to get it. The study doctor must get your authorization (permission) to use or give out any health information that might identify you.

**What protected health information may be used and/or given to others?**

All medical information, including but not limited to information and/or records of any diagnosis or treatment of disease or condition, which may include sexually transmitted diseases (e.g., HIV, etc.) or communicable diseases, drug/alcohol dependency, etc.; all personal identifiers, including but not limited to your name, social security number, medical record number, date of birth, dates of service, etc.; any past, present, and future history, examinations, laboratory results, imaging studies and reports and treatments of any kind, including but not limited to drug/alcohol treatment, psychiatric/psychological treatment; financial/billing information, including but not limited to copies of your medical bills; any other information related to or collected for use in the research study, regardless of whether the information was collected for research or non-research (e.g., treatment) purposes; records about any study drug you received or about study devices used; and consent forms from past studies that might be in your medical record.

**Who may use and give out information about you?**

Information about your health may be used and given to others by the study doctor and staff. They might see the research information during and after the study.

**Who might get this information?**

All Individuals/entities listed in the informed consent document(s), including but not limited to, the physicians, nurses and staff and others performing services related to the research (whether at UAB or elsewhere). Your information may also be given to the sponsor of this research. “Sponsor” includes any persons or companies that are working for or with the sponsor, or are owned by the sponsor, or are providing support to the sponsor (e.g., contract research organization).

Information about you and your health which might identify you may be given to:

• the Office for Human Research Protections (OHRP)

• the U.S. Food and Drug Administration (FDA)

• Department of Health and Human Services (DHHS) agencies

• Governmental agencies in other countries

• Governmental agencies to whom certain diseases (reportable diseases) must be reported

• the University of Alabama at Birmingham - the physicians, nurses and staff working on the research study (whether at UAB or elsewhere); other operating units of UAB, UAB Hospital, UAB Highlands Hospital, University of Alabama Health Services Foundation, Children’s of Alabama, Eye Foundation Hospital, and the Jefferson County Department of Health, as necessary for their operations; the UAB IRB and its staff

• the billing offices of UAB and UAB Health Systems affiliates and/or Children’s of Alabama and its billing agents

• the University of Alabama at Birmingham - the physicians, nurses and staff working on the research study (whether at UAB or elsewhere); other operating units of UAB, UAB Hospital, UAB Highlands Hospital, University of Alabama Health Services Foundation, Children’s of Alabama, Eye Foundation Hospital, and the Jefferson County Department of Health, as necessary for their operations; the UAB IRB and its staff

**Why will this information be used and/or given to others?**

Information about you and your health that might identify you may be given to others to carry out the research study. The sponsor will analyze and evaluate the results of the study. In addition, people from the sponsor and its consultants will be visiting the research site. They will follow how the study is done, and they will be reviewing your information for this purpose.

**What if I decide not to give permission to use and give out my health information?**

By signing this consent form, you are giving permission to use and give out the health information listed above for the purposes described above. If you refuse to give permission, you will not be able to be in this research.

**May I review or copy the information obtained from me or created about me?**

You have the right to review and copy your health information. However, if you decide to be in this study and sign this permission form, you will not be allowed to look at or copy your information until after the research is completed.

**May I withdraw or revoke (cancel) my permission?**

Yes, but this permission will not stop automatically. The use of your personal health information will continue until you cancel your permission. You may withdraw or take away your permission to use and disclose your health information at any time. You do this by sending written notice to the study doctor. If you withdraw your permission, you will not be able to continue being in this study. When you withdraw your permission, no new health information which might identify you will be gathered after that date. Information that has already been gathered may still be used and given to others. This would be done if it were necessary for the research to be reliable.

**Is my health information protected after it has been given to others?**

If you give permission to give your identifiable health information to a person or business, the information may no longer be protected. There is a risk that your information will be released to others. Including others outside of UAB, without your permission.

By participating in this study, you are agreeing to provide the most honest answers you can. Any answers you provide will be anonymized, so that neither the research team nor additional respondents will know which is yours. You will be compensated, provided that you consent to complete the survey and finish before your session expires.

By selecting ‘I agree’, you are consenting to the conditions described above.

- I agree  (1) 

- I disagree  (2) 

End of Block: Consent

Start of Block: Healthcare-Related Experiences

Q4 How many times have you felt mistreated by a medical professional while receiving care, because of your gender identity?

- Never  (1) 

- Once  (2) 

- 2-3 times  (3) 

- 4 or more times  (4) 

Q5 How many times have you feared for your physical safety when you’ve been in a healthcare setting?

- Never  (1) 

- Once  (2) 

- 2-3 times  (3) 

- 4 or more times  (4) 

Q6 How many times have you feared for your mental/emotional wellbeing when you’ve been in a healthcare setting?

- Never  (1) 

- Once  (2) 

- 2-3 times  (3) 

- 4 or more times  (4) 

Q7 Do you currently have a mental health condition or have had a past history of any mental health condition(s)?

- Yes  (1) 

- No  (2) 

Q8 Are there traumas you have experienced in your life that you think your provider should consider when taking care of you?

- Yes  (1) 

- No  (2) 

Display this question:

If Are there traumas you have experienced in your life that you think your provider should consider... = Yes

Q9 If you have experienced in your life that you think your provider should consider when taking care of you, please list them in simple terms:

________________________________________________________________

End of Block: Healthcare-Related Experiences

Start of Block: Sexual and Relationship Experiences

Q10 Has anyone made you have intercourse or oral or anal sex against your will?

- Yes  (1) 

- No  (2) 

Display this question:

If Has anyone made you have intercourse or oral or anal sex against your will? = Yes

Q11 If you were made to have sex against your will, please indicate nature of relationship with that person (e.g., stranger, friend, relative, neighbor, parent, sibling):

________________________________________________________________

Display this question:

If Has anyone made you have intercourse or oral or anal sex against your will? = Yes

Q12 If you were made to have sex against your will, about how many times did that occure in your lifetime?

- Once  (1) 

- 2-3 times  (2) 

- 4 or more times  (3) 

Q13 Other than any incident(s) mentioned above, have there been any other situations in which another person tried to force you to have an unwanted sexual contact?

- Yes  (1) 

- No  (2) 

Display this question:

If Other than any incident(s) mentioned above, have there been any other situations in which another... = Yes

Q14 If there are other situations in which another person tried to force you to have an unwanted sexual contact, about how many times has this happened in your lifetime?

- Once  (1) 

- 2-3 times  (2) 

- 4 or more  (3) 

Q15 Have you participated in transactional sex (also known as sex work, prostitution, etc.)?

- Yes  (1) 

- No  (2) 

Display this question:

If Have you participated in transactional sex (also known as sex work, prostitution, etc.)? = Yes

Q16 If you have participated in transactional sex, about how many times have you done so in your lifetime?

- Once  (1) 

- 2-3 times  (2) 

- 4 or more  (3) 

Display this question:

If Have you participated in transactional sex (also known as sex work, prostitution, etc.)? = Yes

Q17 Are you currently participating in transactional sex?

- Yes, if so, how often?  (1) __________________________________________________

- Maybe  (2) 

- No  (3) 

Q18 Have you been in a relationship (e.g., emotional, sexual, romantic) with someone where you felt there were unequal power dynamics (i.e., one person has more power in the relationship than the other)?

- Yes  (1) 

- No  (2) 

End of Block: Sexual and Relationship Experiences

Start of Block: Crime Related and General Trauma Experiences

Q19 Has anyone attempted to rob or actually robbed you?

- Yes  (1) 

- No  (2) 

Q20 Have you been arrested or incarcerated?

- Yes  (1) 

- No  (2) 

Q21 Do you feel that you have experienced poor treatment by law enforcement services or while being incarcerated?

- Yes  (1) 

- No  (2) 

Q22 Have you seen someone seriously injured or killed?

- Yes  (1) 

- No  (2) 

Q23 Have you had a spouse, romantic partner, child, or other loved one die?

- Yes  (1) 

- No  (2) 

Q24 Have you have had a serious or life-threatening illness?  These 
can be both chronic conditions (e.g., diabetes, HIV, mental health conditions (e.g, anxiety, depression, etc) or acute issues (e.g., heart attacks, cancer, etc) which have impacted your life significantly.

- Yes  (1) 

- No  (2) 

Q25 Has anyone attacked you with a gun, knife, or some other weapon?

- Yes  (1) 

- No  (2) 

Q26 Has anyone attacked you without a weapon?

- Yes  (1) 

- No  (2) 

Q27 Have you ever experienced homelessness?

- Yes  (1) 

- No  (2) 

Q28 Have you ever experienced food insecurity (limited or uncertain access to adequate food)?

- Yes  (1) 

- No  (2) 

Q29 Have you ever experienced unemployment or dire financial struggles?

- Yes  (1) 

- No  (2) 

Q30 Have you ever been in a situation where you were abandoned, disowned, or estranged by members of your biological family?

- Yes  (1) 

- No  (2) 

End of Block: Crime Related and General Trauma Experiences

Start of Block: Gender Dysphoria Experiences

Q31 “Gender dysphoria” describes a sense of unease that a person may have because of a mismatch between their sex assigned at birth and their gender identity.

Q32 Do you experience dysphoria around your sex assigned at birth?

- Yes  (1) 

- No  (2) 

Display this question:

If Do you experience dysphoria around your sex assigned at birth? = Yes

Q33 If you have experienced dysphoria around your sex assigned at birth, please list the circumstances that trigger this dysphoria that you want your provider to know about:

________________________________________________________________

Q34 Does someone misgendering (i.e., referring to you as a gender that you do not identify with) you give you dysphoria?

- Yes  (1) 

- No  (2) 

End of Block: Gender Dysphoria Experiences

Start of Block: Discrimination Experiences

Q35 Answer these questions in relation to discrimination you have faced related to your gender identity.

Q36 How often have you experienced discrimination at school?

- Never  (1) 

- Once  (2) 

- 2-3 times  (3) 

- 4 or more times  (4) 

Q37 How often have you experienced discrimination getting hired or getting a job?

- Never  (1) 

- Once  (2) 

- 2-3 times  (3) 

- 4 or more times  (4) 

Q38 How often have you experienced discrimination at work?

- Never  (1) 

- Once  (2) 

- 2-3 times  (3) 

- 4 or more times  (4) 

Q39 How often have you experienced discrimination getting housing?

- Never  (1) 

- Once  (2) 

- 2-3 times  (3) 

- 4 or more times  (4) 

Q40 How often have you experienced discrimination getting medical care?

- Never  (1) 

- Once  (2) 

- 2-3 times  (3) 

- 4 or more times  (4) 

Q41 How often have you experienced discrimination getting services in a store or restaurant?

- Never  (1) 

- Once  (2) 

- 2-3 times  (3) 

- 4 or more times  (4) 

Q42 How often have you experienced discrimination getting credit, bank loans, or mortgage?

- Never  (1) 

- Once  (2) 

- 2-3 times  (3) 

- 4 or more times  (4) 

Q43 How often have you experienced discrimination on the street or in a public setting?

- Never  (1) 

- Once  (2) 

- 2-3 times  (3) 

- 4 or more times  (4) 

Q44 How often have you experienced discrimination from the police or in the courts?

- Never  (1) 

- Once  (2) 

- 2-3 times  (3) 

- 4 or more times  (4) 

End of Block: Discrimination Experiences

Start of Block: Default Question Block

|  |
| --- |

Q45 What is your age?:

________________________________________________________________

Q46 What is your race? (select one or more):

- White  (1) 

- Black or African American  (2) 

- American Indian/Native American or Alaskan Native  (3) 

- Asian  (4) 

- Native Hawaiian or Pacific Islander  (5) 

- Not Listed, specify:  (6) __________________________________________________

Q47 What is your ethnicity?:

- Hispanic/Latino  (1) 

- Not Hispanic or Latino  (2) 

- Not listed, specify:  (3) __________________________________________________

- Decline to answer  (4) 

Q48 What is your highest level of education:

- Less than high school  (1) 

- High school graduate/GED  (2) 

- Some college or Associate's degree  (3) 

- 4 year degree/Bachelor's degree  (4) 

- Any post-graduate studies (Master's/Doctoral/Professional) degree  (5) 

- Decline to answer  (6) 

Q49 What is your current occupational status (select all that apply):

- Student  (1) 

- Employed, part-time  (2) 

- Employed, full-time  (3) 

- Unemployed  (4) 

- On disability  (5) 

Q50 How would you describe your relationship status?

- Legally or common-law married  (1) 

- Not married but live with a partner  (2) 

- Widowed  (3) 

- Divorced/annulled marriage  (4) 

- Separated  (5) 

- Never married  (6) 

- Other, specify:  (7) __________________________________________________

Q51 Do you currently have health insurance?

- Yes  (1) 

- No  (2) 

Display this question:

If Do you currently have health insurance? = Yes

Q52 What type of health insurance do you have?

- Public (Medicare, Medicaid, VA)  (1) 

- Tricare  (2) 

- Private  (3) 

- Other, specify:  (4) __________________________________________________

Q53 How often do you have a drink containing alcohol?

- Never  (1) 

- Monthly or less  (2) 

- 2-4 times per month  (3) 

- 2-3 times per week  (4) 

- 4 or more times a week  (5) 

Q54 When you do drink, how many standard drinks do you have on a typical day?

- 1 or 2  (1) 

- 3 or 4  (2) 

- 5 or 6  (3) 

- 7 to 9  (4) 

- 10 or more  (5) 

Q55 How often do you have 6 or more drinks on one occasion?

- Never  (1) 

- Less than monthly  (2) 

- Monthly  (3) 

- Weekly  (4) 

- Daily or almost daily  (5) 

Q56 Which of the following illicit/recreational drugs have you used in your lifetime?

- Marijuana/hash  (1) 

- Crack/freebase cocaine  (2) 

- Powder cocaine  (3) 

- Amphetamine/Methamphetamine/Crystal meth  (4) 

- Heroine  (5) 

- Hallucinogens/Club drugs  (6) 

- Prescription drugs in a way not prescribed  (7) 

- Other, please specify:  (8) __________________________________________________

- None/Not applicable  (9) 

Display this question:

If Which of the following illicit/recreational drugs have you used in your lifetime? = Crack/freebase cocaine

And Which of the following illicit/recreational drugs have you used in your lifetime? = Powder cocaine

And Which of the following illicit/recreational drugs have you used in your lifetime? = Amphetamine/Methamphetamine/Crystal meth

And Which of the following illicit/recreational drugs have you used in your lifetime? = Heroine

And Which of the following illicit/recreational drugs have you used in your lifetime? = Hallucinogens/Club drugs

And Which of the following illicit/recreational drugs have you used in your lifetime? = Prescription drugs in a way not prescribed

And Which of the following illicit/recreational drugs have you used in your lifetime? = Other, please specify:

Q57 If you have used illicit/recreational drugs, have you ever injected them?

- Yes  (1) 

- No  (2) 

Q58 Which of the following illicit/recreational drugs do you currently use?

- Marijuana/hash  (1) 

- Crack/freebase cocaine  (2) 

- Powder cocaine  (3) 

- Amphetamine/Methamphetamine/Crystal meth  (4) 

- Heroine  (5) 

- Hallucinogens/Club drugs  (6) 

- Prescription drugs in a way not prescribed  (7) 

- Other, please specify:  (8) __________________________________________________

- None/Not applicable  (9) 

Display this question:

If Which of the following illicit/recreational drugs do you currently use? = Crack/freebase cocaine

And Which of the following illicit/recreational drugs do you currently use? = Powder cocaine

And Which of the following illicit/recreational drugs do you currently use? = Amphetamine/Methamphetamine/Crystal meth

And Which of the following illicit/recreational drugs do you currently use? = Heroine

And Which of the following illicit/recreational drugs do you currently use? = Hallucinogens/Club drugs

And Which of the following illicit/recreational drugs do you currently use? = Prescription drugs in a way not prescribed

And Which of the following illicit/recreational drugs do you currently use? = Other, please specify:

Q59 If you have used illicit/recreational drugs, do you inject any of them?

- Yes  (1) 

- No  (2) 

Q60 Have you ever participated in transactional sex (traded money or drugs for sex)?

- Yes  (1) 

- No  (2) 

- Decline to answer  (3) 

Q61 Do you currently have any of the following mental health conditions? (Mark all that apply):

- Depression  (1) 

- Bipolar Disorder  (2) 

- Anxiety  (3) 

- ADD/ADHD  (4) 

- Schizophrenia/Schizoaffective  (5) 

- PTSD  (6) 

- None or Other, please specify:  (7) __________________________________________________

Q62 What is your sexual orientation?

- Heterosexual  (1) 

- Homosexual  (2) 

- Bisexual  (3) 

- Queer  (4) 

- Not listed, please specify:  (5) __________________________________________________

Q63 What are the genders of your sexual partners? (Mark all that apply):

- Cis Man  (1) 

- Cis Woman  (2) 

- Trans Man  (3) 

- Trans Woman  (4) 

- Non-binary, Genderfluid, Gender diverse  (5) 

- Not listed, please specify:  (6) __________________________________________________

Q64 Please provide the number of sexual partners for the following time-periods:

- Past 30 Days  (1) __________________________________________________

- Lifetime  (2) __________________________________________________

Q65 Have you heard of Pre-Exposure Prophylaxis (PrEP) for HIV (Truvada, etc.)?

- Yes  (1) 

- No  (2) 

Q66 Have you ever been on PrEP in the past?

- Yes  (1) 

- No  (2) 

Q67 Prior to today, which of the following sexually transmitted infections have you had?

- None  (1) 

- Gonorrhea  (2) 

- Chlamydia  (3) 

- Syphilis  (4) 

- Trichomonas  (5) 

- *Mycoplasma genitalium*  (6) 

- HPV/Genital warts  (7) 

- Genital herpes  (8) 

- HIV  (9) 

- Decline to answer  (10) 

- Do not know if I have had any of these  (11) 

Q68 Have you been tested for STIs, including HIV, in the last year?

- Yes  (1) 

- No  (2) 

Q69 Are you currently on hormone therapy?

- Yes  (1) 

- No  (2) 

Q70 Have you had any of the following gender affirming surgeries?  (Mark all that apply):

- Vaginoplasty  (1) 

- Orchiectomy  (2) 

- Breast augmentation  (3) 

- Facial feminization surgery  (4) 

- Vocal cord procedures  (5) 

- Other, please specify:  (6) __________________________________________________

- None/not applicable  (7) 

Q71 Did you use a protective barrier the last time you had sex?

- Yes  (1) 

- No  (2) 

- Do not remember  (3) 

Q72 Do you have a history of receptive anal sex (i.e., bottoming, receiving a penis in your anus during sex)?

- Yes  (1) 

- No  (2) 

Display this question:

If Do you have a history of receptive anal sex (i.e., bottoming, receiving a penis in your anus duri... = Yes

Q73 How often does your partner use condoms during anal sex?

- Always  (1) 

- Sometimes  (2) 

- Never  (3) 

Q74 Have you had at least 1 vaccination to protect against human papillomavirus (HPV)?

- Yes  (1) 

- No  (2) 

- I don't know  (3) 

End of Block: Default Question Block
